# Gout-related inpatient utilization: a study of predictors of outcomes and time trends

**DOI:** 10.1186/s13075-016-0936-y

**Published:** 2016-03-02

**Authors:** Jasvinder A. Singh, Shaohua Yu

**Affiliations:** Medicine Service, Birmingham VA Medical Center, 700 South 19th Street, Birmingham, AL 35233 USA; Department of Medicine at School of Medicine, and Division of Epidemiology at School of Public Health, University of Alabama at Birmingham (UAB), 1705 University Boulevard, Birmingham, AL 35233 USA; Department of Orthopedic Surgery, Mayo Clinic College of Medicine, 200 1st Street SW, Rochester, MN 55905 USA

**Keywords:** Gout, Inpatient utilization, Hospitalization, Comorbidity, Predictors, Length of stay, Hospital discharge, Resource utilization, Charges

## Abstract

**Background:**

To assess inpatient healthcare burden of gout in the USA after an Emergency Department (ED) visit and the predictors of gout-related hospitalizations.

**Method:**

We used the 2009, 2010 and 2012 US National ED Sample (NEDS) data to examine the time trends in inpatient visits with gout as the primary diagnosis. We used the 2012 NEDS data to assess multivariable-adjusted predictors of length of hospital stay, discharge to home (versus other) and total charges for gout-related inpatient visits.

**Results:**

Of the 205,152 ED visits for gout as the primary diagnosis in 2012, 7.7 % resulted in hospitalization. In 2009, 2010 and 2012, 63 %, 63 % and 64.5 % of hospitalized patients were discharged home; respective durations of hospital stay were 4.15, 4.00 and 3.86 days. Older age 50 to <65 years (ref <50), renal failure, heart failure, osteoarthritis and diabetes were associated with a longer hospital stay and self-pay/uninsured status, hospital location in the Midwest or Western USA with a shorter hospital stay for gout. Similar factors were associated with total charges for gout-related admissions. Older age (65 to <80 and ≥80, relative to <50 years), diabetes, self-pay/no charge insurance status, metropolitan area residence, and a longer length of hospital stay were associated with lower odds of discharge to home; and self-pay/no charge (uninsured) status was associated with higher odds of discharge to home, compared to Medicare coverage.

**Conclusions:**

Using a national sample, we noted declining duration of hospital stay and identified factors associated with the length of hospital stay, discharge to home and charges for gout hospitalization following an ED visit. Future studies should examine whether better management of comorbidities in patients with gout can further reduce utilization and cost of gout-related hospitalizations.

## Background

Gout is the most common form of inflammatory arthritis in adults that affects up to 8.3 million Americans [1]; prevalence in European countries is similar at >1 % [2]. In the presence of comorbidities that frequently accompany gout, such as renal failure, heart failure (HF) and hypertension, the treatment of gout can be challenging [3]. Poor quality of gout care has been documented, related to these and other challenges in gout care [4]. Thus, it is not surprising that gout is associated with higher healthcare utilization rates and cost [5].

Previous studies of gout-related utilization focused primarily on costs [6, 7] or Emergency Department (ED) visits [8, 9]. Hospitalization is expensive; 7 % Americans hospitalized in 2012 accounted for 29 % of all healthcare expenses and cost $377 billion [10]. Studies of hospitalization in gout are few (120 citations resulted from a MEDLINE search on 16 December 2015 using the keywords gout and hospitalization), which demonstrates that this area is understudied. Studies have focused on seasonal variation [11], quality of care or specific treatment patterns [12–15], time trends [16], economic burden [16, 17], or comparison of charges in gout versus non-gout population [5, 17]. Several knowledge gaps exist. To our knowledge, none of the previous studies examined predictors of inpatient utilization or disposition after hospitalization for gout. Most studies except one study [16] used non-representative samples from tertiary care centers [12–15] or combined all crystalline diseases [12, 15, 17], which limited the generalizability of findings to general populations with gout.

Thus, there are big knowledge gaps in our understanding of the impact of gout on inpatient healthcare utilization. Our study objective was to address the following key questions using the data from the US National ED Sample (NEDS): (1) which specific comorbidities and patient factors are associated with higher inpatient healthcare utilization, discharge disposition and charges in gout; (2) what is the magnitude and direction of these associations; and (3) whether inpatient utilization due to gout is increasing or decreasing over time.

## Methods

### Data source and study population

We performed this study using the discharge data from the National Emergency Department Sample (NEDS), Healthcare Cost and Utilization Project (HCUP), provided by the Agency for Healthcare Research and Quality (AHRQ) [18]. NEDS is the largest, all-payer US ED database that contains a 20 % stratified sample of ED visits from across the USA [18]. The HCUP State Emergency Department Databases (SEDD) and the State Inpatient Databases (SID) provide data for NEDS [18]. The SEDD and SID capture discharge information on ED visits that do not result versus that result in an admission to the same hospital, respectively. Thus, the denominators for ED-related inpatient admissions (all ED visits) are available in this database. Thirty states, including 950 US hospitals, contributed data in 2012. NEDS contains event-level data. NEDS provides appropriate weights to obtain weighted national estimates. In 2012, 31 million ED visits were weighted to calculate the national estimates related to 134 million ED visits in the USA [18]. NEDS is publicly available. For this study, we limited analyses to hospitalization with gout as the primary diagnosis in those who had gout-related ED visits. We identified gout-related visits using the International Classification of Diseases, Ninth revision, Common Modification (ICD-9-CM) code of 274.xx in the respective visit (a code listed as primary for the index ED visit for ED-visit counts and for the index inpatient visit for inpatient visit counts), an approach shown to be valid previously [19]. The Institutional Review Board at the University of Alabama at Birmingham approved the study and waived the need for written informed consent for this database study.

### Outcomes of interest and covariates

Study outcomes of interest in patients hospitalized with gout as the primary diagnosis after an ED visit were: (1) duration of hospital stay; (2) discharge to home; and (3) total charges (ED and inpatient).

In addition to the reasons for ED visit (diagnoses and procedures), NEDS includes other important patient/hospital characteristics, such as age, sex, insurance status, residence (urban versus rural), and the annual median household income estimated using residential zip code. Hospital characteristics include geographical region, location in metropolitan or non-metropolitan area, and whether the hospital is teaching versus non-teaching. For each NEDS visit, up to 15 ICD-9-CM diagnostic codes, nine ICD-9-CM procedures and 15 additional procedures coded using Current Procedural Terminology (CPT) are provided, which we used to assess comorbidities.

### Statistical analysis

We used 2009, 2010 and 2012 data to examine time trends in the length of hospital stay and the proportion of patients discharged to home, since 2011 data had data duplication issues and were not available from AHRQ at the time of study conduct (https://www.hcup-us.ahrq.gov/db/nation/neds/2011NEDSErrataNotification022415.pdf). We used the 2012 NEDS data (most recent data available) to analyze whether patient and hospital factors were associated with outcomes following inpatient admission with gout as the primary diagnosis after an ED visit (disposition to home; length of hospital stay; total hospital charges (ED plus inpatient)). We included prespecified patient and hospital characteristics listed in covariate section (see paragraph above) as potential predictors. We performed multivariable-adjusted logistic regression (discharge disposition) or linear regression (charges, length of stay) using SAS version 9.1 (SAS Institute, Inc., Cary, NC, USA). Analyses were performed for log odds of charges and duration of hospital stay due to their skewed distribution; the log transformation of each variable showed normal distribution.

## Results

### Patient characteristics

Of the 205,152 ED visits for gout as the primary diagnosis in 2012, 7.7 % resulted in a hospital admission (Table 1). Characteristics of patients with gout-related ED visit (overall), as well by whether an ED visit resulted in a hospitalization or not are provided (Table 1). Those who were admitted to the hospital were more likely to be female, older, living in a metropolitan area, or have higher household income, have Medicare as primary payer, more likely to have comorbidities and were seen at a hospital located in the Northeast or seen at a Metropolitan, teaching hospital (Table 1).

**Table 1:** Demographic characteristics for 2012 NEDS study population

	2012 NEDS (all)	2012 NEDS, not admitted	2012 NEDS who were admitted	*P* value, not admitted vs. admitted
	N = 205,152	N = 189,255	N = 15,870	
Age, in years				
mean (SE)	55.44 (0.16)	54.50 (0.16)	66.66 (0.35)	<0.0001
Sex				
Female	46,839 (22.83)	41,818 (22.10)	5,021 (31.64)	<0.0001
Patient location (residence)				<0.0001
Micropolitan/not metro	47,371 (23.19)	45,563 (24.17)	1,808 (11.48)	
Metropolitan (large or small)	156,905 (76.81)	142,958 (75.83)	13,948 (88.52)	
Median household income				<0.0001
1st quartile (<$38,999)	81,588 (40.71)	76,396 (41.23)	5,192 (33.47)	
2nd quartile ($39,000 to $47,999)	48,791 (24.35)	45,224 (24.46)	3,567 (23.00)	
3rd quartile ($48,000 to $62,999)	40,462 (20.19)	37,054 (20.04)	3,409 (21.98)	
4th quartile ($63,000 or more)	29,554 (14.75)	26,211 (14.18)	3,343 (21.55)	
Primary payer				<0.0001
Medicare	72,568 (35.41)	62,733 (33.18)	9,835 (62.02)	
Medicaid	27,556 (13.45)	25,773 (13.63)	1,783 (11.25)	
Private insurance	51,329 (25.05)	48,730 (25.77)	2,599 (16.39)	
Self-pay/no charge	45,241 (22.07)	44,068 (23.30)	1,173 (7.40)	
Other	8,254 (4.03)	7,788 (4.12)	466 (2.94)	
Hospital region				<0.0001
Northeast	35,976 (17.54)	31,314 (16.54)	4,661 (29.37)	
Midwest	40,729 (19.85)	37,434 (19.78)	3,295 (20.76)	
South	97,904 (47.72)	92,088 (48.65)	5,817 (36.65)	
West	30,543 (14.89)	28,446 (15.03)	2,097 (13.21)	
Teaching status of hospital				<0.0001
Metropolitan non-teaching or non-metro	125,106 (60.98)	118,362 (62.53)	6,744 (42.49)	
Metropolitan teaching	80,047 (39.02)	70,902 (37.47)	9,126 (57.51)	
Comorbidities				
Coronary heart disease	13,548 (6.60)	9436 (4.99)	4,112 (25.91)	<0.0001
Hyperlipidemia	23,862 (2.94)	17,829 (9.42)	6,033 (38.02)	<0.0001
Renal failure	13,176 (6.42)	6,500 (3.43)	6,676 (42.07)	<0.0001
Heart failure	10,029 (4.89)	6,455 (3.41)	3,574 (22.52)	<0.0001
Hypertension	84,352 (41.12)	71,709 (37.88)	12,644 (79.67)	<0.0001
Diabetes	32,774 (15.98)	26,653 (9.42)	6,121 (38.02)	<0.0001
COPD	5,487 (2.67)	3,714 (1.96)	1,773 (11.17)	<0.0001
Osteoarthritis	6,755 (3.29)	4,253 (2.25)	2,502 (15.77)	<0.0001

*NEDS* National Emergency Department Sample, *SE* standard error, *COPD* chronic obstructive pulmonary disease

Duration of hospital stay with gout as the primary diagnosis seemed to diagnosis decreased significantly over time, from 4.15 days in 2009 to 3.86 days in 2012, which was significant (Table 2; *p* <0.01 to *p* <0.001 on a paired *t* test for varying assumption of correlation coefficient ranging from 0.80 to −0.80, respectively). This corresponded to a decrease of duration of hospital stay by 6.99% over 4 years or a reduction by 1.7 %/year roughly. The observations were similar for gout as primary or secondary diagnosis. Respectively, 63 %, 63 % and 64.5 % patients with a primary diagnosis of gout and 57.7 %, 57.7 % and 57.2 % for those with gout as primary or secondary diagnosis were discharged home from the hospital, in years 2009, 2011 and 2012, respectively (Table 2).

**Table 2:** Outcomes of patients with a hospital admission for gout after an Emergency Department (ED) visit

	2009 NEDS	2010 NEDS	2012 NEDS
Duration of hospital stay, in days			
Gout as primary diagnosis for ED visit, mean (SE)	4.15 (0.08)	4.00 (0.07)	3.86 (0.06)
Gout primary or secondary diagnosis for ED visit, mean (SE)	4.96 (0.05)	4.81 (0.04)	4.69 (0.05)
Hospitalization disposition for gout as as the primary diagnosis, n (%)			
Discharged home	9,634 (63.02)	10,999 (63.22)	10,232 (64.47)
Skilled nursing facility, intermediate care facility, or another type of facility	2,934 (19.19)	3,188 (18.33)	2,835 (17.86)
Transferred to short-term hospital	104 (0.68)	148 (0.85)	92 (0.58)
Home health care	2,447 (16.00)	2,908 (16.71)	2,556 (16.10)
Against medical advice	118 (0.77)	115 (0.66)	134 (29.82)
Died	52 (0.34)	39 (0.23)	22 (0.14)
Hospitalization disposition with gout as the primary or secondary diagnosis^*^, n (%)			
Discharged home	257,348 (57.72)	272,281 (57.75)	285,080 (57.22)
Skilled nursing facility, intermediate care facility, and another type of facility	94,314 (21.15)	98,853 (20.97)	104,135 (20.90)
Transferred to short-term hospital	12,241 (2.75)	12,241 (2.60)	13,206 (2.65)
Home healthcare	68,116 (15.28)	75,237 (15.96)	81,885 (16.44)
Against medical advice	3,524 (0.79)	3,516 (0.75)	4,198 (0.84)
Died	10,069 (2.26)	9,133 (1.94)	9,547 (1.92)

*NEDS* National Emergency Department Sample, *ED* emergency department, *SE* standard error

*Statistics for hospitalizations for which gout was either primary or secondary diagnosis

### Predictors of the length of hospital stay, discharge disposition and total charges in patients hospitalized with gout as primary diagnosis

In multivariable-adjusted linear regression analyses, age 50 to <65 years (compared to <50 years) and the presence of renal failure, heart failure, diabetes or osteoarthritis were associated with a longer hospital stay for a hospitalization due to gout (Table 3). In contrast, self-pay/no charge (uninsured) or private insurance status, and hospital location in Midwest or Western USA, were associated with a shorter hospital stay for a gout hospitalization (Table 3).

**Table 3:** Predictors of log of duration of hospital stay among patients with gout who were admitted to the hospital after presenting to the Emergency Department (ED) with gout using linear regression

	Univariate		Multivariable-adjusted	
	B-estimate (95 % CI)	*P* value	B-estimate (95 % CI)	*P* value
Age				
<50	Ref		Ref	
50 to <65	**0.12 (0.07, 0.17)**	**<0.0001**	**0.07 (0.02, 0.12)**	**0.0081**
65 to <80	**0.20 (0.14, 0.25)**	**<0.0001**	**0.07 (0.01, 0.14)**	**0.0287**
≥80	**0.22 (0.16, 0.27)**	**<0.0001**	**0.09 (0.01, 0.17)**	**0.0234**
Gender				
Female	Ref		Ref	
Male	**−0.08 (−0.12, −0.04)**	**<0.0001**	−0.04 (−0.08, 0.00)	0.0518
Median household income				
1st quartile	Ref		Ref	
2nd quartile	−0.01 (−0.06, 0.03)	0.5966	−0.01 (−0.05, 0.04)	0.7384
3rd quartile	−0.03 (−0.09, 0.02)	0.2095	−0.03 (−0.08, 0.03)	0.3354
4th quartile	−0.01 (−0.06, 0.04)	0.6627	−0.02 (−0.07, 0.03)	0.4458
Primary payer				
Medicare	Ref		Ref	
Medicaid	**−0.09 (−0.16, −0.02)**	**0.0093**	−0.05 (−0.12, 0.03)	0.2501
Private insurance	**−0.14 (−0.19, −0.09)**	**<0.0001**	**−0.07 (−0.13, −0.01)**	**0.02**
Self-pay/no charge	**−0.25 (−0.31, −0.19)**	**<0.0001**	**−0.16 (−0.23, −0.09)**	**<0.0001**
Other	**−0.17 (−0.26, −0.08)**	**0.0003**	−0.08 (−0.18, 0.02)	0.1264
Patient location (residence)				
Micropolitan/not metro	Ref		Ref	
Metro (large or small)	0.02 (−0.05, 0.08)	0.6063	0.02 (−0.05, 0.09)	0.4927
Hospital region				
Northeast	Ref		Ref	
Midwest	**−0.13 (−0.18, −0.07)**	**<0.0001**	**−0.12 (−0.18, −0.07)**	**<0.0001**
South	−0.03 (−0.09, 0.02)	0.2100	−0.02 (−0.08, 0.03)	0.4363
West	**−0.14 (−0.21, −0.08)**	**<0.0001**	**−0.11 (−0.18, −0.04)**	**0.0025**
Teaching status of hospital				
Metropolitan non-teaching or non-metro	Ref		Ref	
Metropolitan teaching	0.03 (−0.01, 0.07)	0.1344	0.01 (−0.03, 0.06)	0.5442
Comorbidities				
CHD (ref: no)	**0.06 (0.02, 0.10)**	**0.0018**	0.01 (−0.04, 0.05)	0.8225
Hyperlipidemia (ref: no)	−0.00 (−0.04, 0.03)	0.9381	−0.04 (−0.08, 0.01)	0.258
Renal failure (ref: no)	**0.13 (0.09, 0.17)**	**<0.0001**	**0.10 (0.06, 0.14)**	**<0.0001**
Heart failure (ref: no)	**0.14 (0.10, 0.17)**	**<0.0001**	**0.08 (0.04, 0.12)**	**0.0002**
Hypertension (ref: no)	0.04 (−0.00, 0.08)	0.0832	−0.04 (−0.08, 0.01)	0.0938
Diabetes (ref: no)	**0.09 (0.05, 0.13)**	**<0.0001**	**0.05 (0.01, 0.09)**	**0.0078**
COPD (ref: no)	0.04 (−0.02, 0.09)	0.2023	−0.02 (−0.08, 0.04)	0.4902
Osteoarthritis (ref: no)	**0.08 (0.03, 0.12)**	**0.0027**	**0.06 (0.01, 0.11)**	**0.0250**

Significant beta coefficients are in bold

*CI* confidence interval, *Ref* reference category, *CHD* coronary heart disease, *COPD* chronic obstructive pulmonary disease

For interpretation in numeric terms for hospital stay, the coefficients from this regression with log (hospital stay) as an outcome should be transformed as e^x,^ where x = beta-estimate. For example, compared to age <50, hospital stay for ages 50 to <65 was 1.07 times higher (beta coefficient = 0.07; e^0.07^ = 1.07). On the other hand, compared to Medicare, private insurance was associated with 0.93 times (beta coefficient = −0.07; e^−0.07^ = 0.93) and self-pay/no charge with 0.85 times (beta coefficient = −0.16; e^−0.16^ = 0.85), the duration of hospital stay. Positive beta-coefficients in this regression with log outcome indicate a longer length of stay and negative beta-coefficients indicate a shorter length of stay. Patients residing in the Midwest had 0.89 times and in the West had 1.57 times the duration of hospital stay (reference, Northeast). Compared to patients without each condition, patients with renal failure had 1.10 times, heart failure, 1.08 times, diabetes, 1.05 times and osteoarthritis, 1.06 times, the duration of hospital stay

In multivariable-adjusted analyses, older age (65 to <80 and ≥80, relative to <50 years), patient residence in metropolitan area, concomitant diabetes, and a longer length of hospital stay were associated with significantly lower odds of discharge to home (Table 4); self-pay/no charge (uninsured) status was associated with higher odds of discharge to home, compared to Medicare coverage.

**Table 4:** Predictors of discharge to home (reference, non-home discharge) among patients who had a hospital admission after presenting to the Emergency Department (ED) with gout using logistic regression

	Univariate		Multivariable-adjusted	
	Odds ratio (95 % CI)	*P* value	Odds ratio (95 % CI)	*P* value
Age				
<50	Ref			
50 to <65	**0.53 (0.38, 0.73)**	**0.0001**	0.69 (0.47, 1.01)	0.0573
65 to <80	**0.15 (0.11, 0.20)**	**<0.0001**	**0.25 (0.16, 0.37)**	**<0.0001**
≥80	**0.08 (0.06, 0.11)**	**<0.0001**	**0.13 (0.09, 0.20)**	**<0.0001**
Gender				
Female	Ref		Ref	
Male	**1.81 (1.55, 2.12)**	**<0.0001**	1.16 (0.97, 1.39)	0.1128
Median household income				
1st quartile (<$38,999)	Ref		Ref	
2nd quartile ($39,000 to $47,999)	1.05 (0.85, 1.31)	0.6413	1.12 (0.87, 1.44)	0.3930
3rd quartile ($48,000 to $62,999)	0.89 (0.70, 1.12)	0.2998	1.02 (0.77, 1.36)	0.8844
4th quartile ($63,000 or more)	0.81 (0.64, 1.01)	0.0618	1.09 (0.84, 1.42)	0.5083
Primary payer				
Medicare	Ref		Ref	
Medicaid	**3.82 (2.92, 5.00)**	**<0.0001**	1.49 (1.00, 2.24)	0.0527
Private insurance	**3.28 (2.62, 4.11)**	**<0.0001**	1.29 (1.00, 1.67)	0.0523
Self-pay/no charge	**11.42 (7.30, 17.85)**	**<0.0001**	**2.77 (1.54, 4.96)**	**0.0006**
Other	**4.59 (2.50, 8.42)**	**<0.0001**	1.49 (0.79, 2.81)	0.2218
Patient location (residence)				
Micropolitan/not metro	Ref		Ref	
Metropolitan (large or small)	0.84 (0.67, 1.07)	0.1589	**0.72 (0.54, 0.97)**	**0.0282**
Hospital region				
Northeast	Ref		Ref	
Midwest	1.17 (0.89, 1.54)	0.2682	0.98 (0.73, 1.31)	0.8700
South	**1.28 (1.00, 1.62)**	**0.0477**	1.20 (0.90, 1.59)	0.2138
West	1.58 (1.17, 2.13)	0.0028	1.24 (0.91, 1.69)	0.1821
Teaching status of hospital				
Metropolitan non-teaching or non-metro	Ref		Ref	
Metropolitan teaching	1.04 (0.86, 1.24)	0.7066	1.09 (0.89, 1.34)	0.3909
Comorbidities				
Coronary heart disease (ref: no)	**0.58 (0.48, 0.69)**	**<0.0001**	0.94 (0.75, 1.16)	0.5433
Hyperlipidemia (ref: no)	0.89 (0.78, 1.03)	0.1152	1.18 (0.99, 1.39)	0.0604
Renal failure (ref: no)	**0.61 (0.52, 0.73)**	**<0.0001**	0.87 (0.71, 1.06)	0.1528
Heart failure (ref: no)	**0.52 (0.44, 0.61)**	**<0.0001**	0.88 (0.72, 1.07)	0.2002
Hypertension (ref: no)	**0.77 (0.64, 0.93)**	**0.0050**	1.09 (0.86, 1.39)	0.4554
Diabetes (ref: no)	**0.68 (0.58, 0.78)**	**<0.0001**	**0.78 (0.64, 0.95)**	**0.0121**
COPD (ref: no)	**0.58 (0.46, 0.72)**	**<0.0001**	0.79 (0.60, 1.04)	0.0871
Osteoarthritis (ref: no)	**0.60 (0.49, 0.74)**	**<0.0001**	0.85 (0.68, 1.06)	0.1414
Length of stay, in days (per day increase)	**0.79 (0.76, 0.83)**	**<0.0001**	**0.80 (0.76, 0.83)**	**<0.0001**

Significant odds ratios are in bold

*CI* confidence interval, *Ref* reference category, *COPD* chronic obstructive pulmonary disease

In multivariable-adjusted analyses, older age 50 to <65 (relative to <50 years), metropolitan area residence, Western USA hospital location and the presence of renal failure or heart failure were associated with higher total charges in hospitalized patients, while those with “other” primary payer (compared to Medicare coverage) had lower total hospital charges (Table 5).

**Table 5:** Predictors of log of inpatient hospital charges among patients with gout who were admitted to the hospital after presenting to the Emergency Department (ED) with gout using linear regression

	Univariate		Multivariable-adjusted	
	B-estimate (95 % CI)	*P* value	B-estimate (95 % CI)	*P* value
Age				
<50	Ref		Ref	
50– <65	**0.11 (0.03, 0.19)**	**0.0055**	**0.10 (0.02, 0.18)**	**0.0129**
65– <80	**0.13 (0.06, 0.21)**	**0.0009**	0.06 (−0.03, 0.16)	0.1681
≥80	**0.13 (0.05, 0.21)**	**0.0024**	0.04 (−0.07, 0.14)	0.4682
Gender				
Female	Ref		Ref	
Male	−0.02 (−0.07, 0.03)	0.4747	−0.02 (−0.07, 0.03)	0.4667
Median household income				
1st quartile (<$38,999)	Ref		Ref	
2nd quartile (39,000 to 47,999)	0.02 (−0.09, 0.12)	0.7360	−0.02 (−0.12, 0.08)	0.7445
3rd quartile (48,000 to 62,999)	0.09 (−0.03, 0.20)	0.1558	0.01 (−0.11, 0.13)	0.8524
4th quartile ($63,000 or more)	0.10 (−0.05, 0.26)	0.1891	0.00 (−0.17, 0.16)	0.9868
Primary payer				
Medicare	Ref		Ref	
Medicaid	−0.04 (−0.15, 0.06)	0.4274	−0.05 (−0.16, 0.06)	0.3690
Private insurance	**−0.11 (−0.18, −0.03)**	**0.0090**	−0.07 (−0.16, 0.02)	0.1432
Self-pay/no charge	−0.11 (−0.21, 0.00)	0.0851	−0.05 (−0.17, 0.07)	0.4134
Other	−0.15 (−0.3, 0.02)	0.0814	**−0.25 (−0.42, −0.08)**	**0.0040**
Patient location (residence)				
Micropolitan/not metro	Ref		Ref	
Metro (large or small)	**0.23 (0.12, 0.35)**	**0.0001**	**0.22 (0.11, 0.33)**	**0.0001**
Hospital region				
Northeast	Ref		Ref	
Midwest	−0.13 (−0.32, 0.06)	0.1889	−0.13 (−0.33, 0.07)	0.2064
South	−0.13 (−0.33, 0.08)	0.2283	−0.11 (−0.33, 0.11)	0.3367
West	**0.40 (0.18, 0.61)**	**0.0003**	**0.45 (0.24, 0.66)**	**<0.0001**
Teaching status of hospital				
Metropolitan non-teaching or non-metro	Ref		Ref	
Metropolitan teaching	0.01 (−0.12, 0.15)	0.8413	0.00 (−0.13, 0.13)	0.9667
Comorbidities				
CHD (ref: no)	**0.10 (0.03, 0.16)**	**0.0053**	0.04 (−0.03, 0.11)	0.2616
Hyperlipidemia (ref: no)	−0.02 (−0.07, 0.03)	0.4388	−0.05 (−0.10, 0.00)	0.0618
Renal failure (ref: no)	**0.15 (0.09, 0.22)**	**<0.0001**	**0.12 (0.07, 0.18)**	**<0.0001**
Heart failure (ref: no)	**0.16 (0.10, 0.23)**	**<0.0001**	**0.13 (0.06, 0.19)**	**0.0001**
Hypertension (ref: no)	−0.00 (−0.07, 0.07)	0.9331	−0.05 (−0.12, 0.03)	0.2028
Diabetes (ref: no)	**0.08 (0.02, 0.13)**	**0.0058**	0.04 (−0.02, 0.10)	0.1590
COPD (ref: no)	**0.10 (0.02, 0.18)**	**0.0167**	0.06 (−0.02, 0.14)	0.1744
OA (ref: no)	0.01 (−0.07, 0.09)	0.7755	0.02 (−0.05, 0.10)	0.5026

Significant odds ratios are in bold

*CI* confidence interval, *Ref* reference category, *CHD* coronary heart disease, *COPD* chronic obstructive pulmonary disease, *OA* osteoarthritis

For interpretation in numeric terms for hospital charges, the coefficients from this regression with log (hospital charges) as an outcome should be transformed as e^x,^ where x = beta-estimate. For example, compared to age <50, hospital charges for ages 50 to <65 was 1.10 times higher (beta coefficient = 0.10; e^0.10^ = 1.10). On the other hand, compared to Medicare, “other” insurance was associated with 0.78 times the duration of hospital stay (beta coefficient =− 0.25; e^−0.25^ = 0.78). Positive beta coefficients in this regression with log outcome indicate higher charges and negative beta coefficients indicate lower hospital charges. Patients residing in metropolitan area had hospital charges 1.25 times (reference, non-metro) and in the West 1.57 times hospital charges (reference, Northeast); patients with renal failure had 1.12 times charges and with heart failure, 1.24 times hospital charges

## Discussion

Our study of hospitalization with gout as the primary diagnosis after an ED visit using a national US sample provides an understanding of the predictors of healthcare and economic burden of gout in the USA and the time trends in hospitalizations due to gout. Several findings are novel and merit further discussion.

Time trends were noted in the length of gout-related hospitalization. The length of hospital stay decreased by 1.7 %/year. This decline was similar to that noted for acute myocardial infarction at 1.8 %/year from 2001 to 2011 [20] and knee/hip arthroplasty at 1.5 %/year from 2003 to 2010 [21], but larger than the 0.2 % annual reduction in length of stay for all hospitalizations in the USA from 2003 to 2012 [22]. Availability of two new urate-lowering therapies since 2009 (febuxostat and pegloticase) may have contributed to this greater reduction. We are unaware of any national quality improvement campaigns for hospitalized gout patients over this time period, or favorable national trends in quality of gout care in outpatient setting that could lead to this decrease. The database did not have any data on inpatient gout quality indicators or medication use, and therefore we were unable to assess this directly using the database. We caution that, even though we found that the difference between 2009 and 2012 hospitalization length of stay was significant and we calculated it based on a wide range of correlation coefficients (+0.80 to −0.80), a more appropriate test for the time trend is repeated measurement mixed model analysis, which could not be performed due to the non-availability of patient-level data.

We identified several patient and hospital characteristics that were significantly associated with the duration of hospital stay, discharge to home and total hospital charges. The study of factors associated with discharge to home for gout-related hospitalization adds to the current knowledge. We found that self-pay/uninsured status was associated with a higher likelihood and older age, metropolitan area residence, diabetes and a longer length of hospital stay were associated with a lower likelihood of discharge to home. In the absence of previous studies of predictors of discharge disposition of patients hospitalized for gout, these findings offer a new insight. Financial burden for self-pay patients and better social support for patients in non-metropolitan areas [23] may be the respective reasons for the higher likelihood of discharge to home in these patients. Recognition that these characteristics associated with discharge disposition can now allow identification of high-risk patients, when they are admitted to the hospital with gout. Older age is associated with nursing home placement due to higher burden of medical illnesses [24], therefore it is not surprising to see it inversely related to discharge to home. Involvement of social services and early discharge planning, especially in those at the highest risk, might further increase the proportion of hospitalized gout patients discharged to home.

Diabetes was associated with nursing home admission in patients hospitalized for heart failure [25]. Our study finding that diabetes is associated with lower odds of discharge to home extends this finding to patients with gout. Diabetes is associated with a decline in mobility and functional status [26]. Limitation of mobility is a risk factor for discharge to non-home settings after hospitalization [24]. This might explain the impact of diabetes on post-hospitalization discharge disposition. Future studies should also examine as to whether specific aspects of diabetes (complications such as neuropathy/nephropathy/retinopathy versus amyotropy versus current blood glucose levels) are responsible for this association.

We also found that several (but not all) comorbidities, namely heart failure, renal failure, diabetes and osteoarthritis, were associated with a longer hospital stay in patients admitted for gout for after an ED visit. For an easier interpretation of regression coefficients for the duration of hospital stay and hospital charges, we added table legends that provides the reader with transformation of the beta coefficients (i.e., exponentiation); a negative coefficient in these tables assessing log of the outcome variable translates to <1 multiple, and a positive coefficient to >1 multiple, compared to the reference category. We hypothesize that treatments frequently used in patients admitted with acute gout, may contribute to worsening of renal function or heart failure, which in turn can prolong the hospital stay. These include corticosteroid use associated with sodium and fluid retention [27–29] that can worsen hypertension and potentially heart failure; non-steroidal anti-inflammatory drugs (NSAIDs) that can lead to new onset/worsening renal function in patients with pre-existing chronic renal failure, diabetic nephropathy and/or heart failure [30–32]; and NSAID-associated fluid retention that can worsen heart failure and renal failure [27]. Use of lower doses of NSAIDs or corticosteroids to avoid worsening of renal or heart failure with full doses (due to relative contraindications), as suggested (but not endorsed) in the 2012 American College of Rheumatology (ACR) gout guideline [33], may also lead to a longer time to resolve a gout flare in these patients, and hence a longer hospital stay. These comorbidities are associated with a higher risk of gout flares [34], and it is possible that gout flares are more severe in patients with these concomitant comorbidities, which requires a longer hospital stay for optimal treatment and resolution.

Whether osteoarthritis-associated symptoms worsen during acute gout flares is not well described. This is possible, and may be contributing to a longer hospitalization in these patients. Chronic disability and functional limitation associated with osteoarthritis may also contribute to a longer hospitalization in these patients with gout. Whether a shorter hospital stay associated with certain hospital and insurance characteristics, such as self-pay/uninsured status, Midwest or West US location, is due to practice pattern variations or other reasons, needs to be examined in future studies. Factors associated with higher total charges were similar to that noted for length of hospital stay, which adds to the consistency of findings. It remains to be seen, whether triage pathways or targeted interventions that optimize these comorbidities in high-risk gout patients can improve the outcomes and reduce healthcare utilization and cost.

Our study has several limitations. We examined ED-associated inpatient utilization due to gout, the main objective of our study. Some hospitalizations may occur from non-ED settings and our study findings are likely not generalizable to those, since they are likely to differ in outcomes. NEDS counts visits, not patients, therefore it is not possible at present to assess the occurrence of repeat visits by the same patients to the ED and factors associated with this phenomenon; these sort of analyses could provide great insights into gout-related ED utilization. NEDS does not provide laboratory or pharmacy costs, which would allow a more complete assessment of resource utilization. NEDS provides data on charges, not actual cost to the hospital or the actual expense paid by the insurer. The charges and the costs/expenses can differ slightly or by a significant margin, depending on how much the charges are inflated relative to the cost of services. The non-availability of the 2011 data due to data duplication issues limited our ability to do detailed time trend analyses, a secondary study objective. The availability of these would have informed time-trend analyses. There was no impact of these missing 2011 data on the main analyses related to predictors of discharge to home, charges and the length of hospital stay, since analyses were performed on the data from the most recent year, i.e., 2012.

Our study has several strengths. The NEDS allowed us to produce national estimates about ED-related inpatient visits due to gout in the USA, given the weighting scheme provided. Its large sample size and representativeness allows study of uncommon/rare outcomes and generalizability of study findings. Several common comorbidities seen in gout, such as heart disease, renal failure and osteoarthritis were adjusted, which make these estimates relevant for gout patients. Findings were robust with a great similarity of factors associated with length of hospital stay and charges.

## Conclusions

In conclusion, our study provides US estimates of healthcare utilization and burden related to hospitalization with gout as the primary diagnosis after an ED visit. We found that several comorbidities and patient and hospital characteristics were associated with a lower risk of discharge to home, a longer hospital stay and higher total charges in patients with gout. Findings from our study should lead to further research into modifiable predictors of outcomes of gout-associated hospitalization. This advance in knowledge can spur the development of potential interventions to reduce inpatient healthcare utilization and costs due to gout in settings and optimize outcomes.

### IRB approval

The University of Alabama at Birmingham’s Institutional Review Board approved this study and all investigations were conducted in conformity with ethical principles of research.

## Abbreviations

AHRQ: Agency for Healthcare Research and Quality
ED: Emergency Department
HCUP: Healthcare Cost and Utilization Project
HF: Heart failure
ICD-9-CM: International Classification of Diseases, Ninth revision, Common Modification
NEDS: National Emergency Department Sample
NSAIDs: Non-steroidal anti-inflammatory drugs
SEDD: State Emergency Department Databases
SID: State Inpatient Databases

## References

[1] Zhu Y, Pandya BJ, Choi HK (2011). Prevalence of gout and hyperuricemia in the US general population: the National Health and Nutrition Examination Survey 2007 –2008. Arthritis Rheum..

[2] Annemans L, Spaepen E, Gaskin M, Bonnemaire M, Malier V, Gilbert T (2008). Gout in the UK and Germany: prevalence, comorbidities and management in general practice 2000 –2005. Ann Rheum Dis..

[3] Rees F, Hui M, Doherty M (2014). Optimizing current treatment of gout. Nat Rev Rheumatol..

[4] Singh JA, Hodges JS, Toscano JP, Asch SM (2007). Quality of care for gout in the US needs improvement. Arthritis Rheum..

[5] Wu EQ, Forsythe A, Guerin A, Yu AP, Latremouille-Viau D, Tsaneva M (2012). Comorbidity burden, healthcare resource utilization, and costs in chronic gout patients refractory to conventional urate-lowering therapy. Am J Ther..

[6] Saseen JJ, Agashivala N, Allen RR, Ghushchyan V, Yadao AM, Nair KV (2012). Comparison of patient characteristics and gout-related health-care resource utilization and costs in patients with frequent versus infrequent gouty arthritis attacks. Rheumatology..

[7] Park H, Rascati KL, Prasla K, McBayne T (2012). Evaluation of health care costs and utilization patterns for patients with gout. Clin Ther..

[8] Singh JA, Sarkin A, Shieh M, Khanna D, Terkeltaub R, Lee SJ (2011). Health care utilization in patients with gout. Semin Arthritis Rheum..

[9] Garg R, Sayles HR, Yu F, Michaud K, Singh J, Saag KG (2013). Gout-related health care utilization in US emergency departments, 2006 through 2008. Arthritis Care Res..

[10] Moore B, Levit K, Elixhauser A. Costs for hospital stays in the United States, 2012. HCUP Statistical Brief #181. October 2014. Rockville: Agency for Healthcare Research and Quality. http://www.hcup-us.ahrq.gov/reports/statbriefs/sb181-Hospital-Costs-United-States-2012.pdf. 25521003

[11] Karmacharya P, Pathak R, Aryal MR, Giri S, Donato AA. Seasonal variation in acute gouty arthritis: data from Nationwide Inpatient Sample. Clin Rheumatol. 2015. Epub ahead of print. 10.1007/s10067-015-3042-7 26255189

[12] Kamalaraj N, Gnanenthiran SR, Kathirgamanathan T, Hassett GM, Gibson KA, McNeil HP (2012). Improved management of acute gout during hospitalization following introduction of a protocol. Int J Rheum Dis..

[13] Petersel D, Schlesinger N (2007). Treatment of acute gout in hospitalized patients. J Rheumatol..

[14] Jackson G, Dalbeth N, Te Karu L, Winnard D, Gow P, Gerard C (2014). Variation in gout care in Aotearoa New Zealand: a national analysis of quality markers. N Z Med J..

[15] Teichtahl AJ, Clemens L, Nikpour M, Romas E (2014). A prospective study of acute inpatient gout diagnoses and management in a tertiary hospital: the determinants and outcome of a rheumatology consultation. Intern Med J..

[16] Robinson PC, Merriman TR, Herbison P, Highton J (2013). Hospital admissions associated with gout and their comorbidities in New Zealand and England 1999 –2009. Rheumatology (Oxford).

[17] Maravic M, Ea HK (2015). Hospital burden of gout, pseudogout and other crystal arthropathies in France. Joint Bone Spine..

[18] Introduction to the HCUP Nationwide Emergency Department Sample (NEDS) 2012. Rockville: Agency for Healthcare Research and Quality. http://www.hcup-us.ahrq.gov/db/nation/neds/NEDS_Introduction_2012.jsp.

[19] Singh JA (2013). Veterans Affairs databases are accurate for gout-related health care utilization: a validation study. Arthritis Res Ther..

[20] Sugiyama T, Hasegawa K, Kobayashi Y, Takahashi O, Fukui T, Tsugawa Y (2015). Differential time trends of outcomes and costs of care for acute myocardial infarction hospitalizations by ST elevation and type of intervention in the United States, 2001 –2011. J Am Heart Assoc..

[21] Cram P, Lu X, Kates SL, Singh JA, Li Y, Wolf BR (2012). Total knee arthroplasty volume, utilization, and outcomes among Medicare beneficiaries, 1991 –2010. JAMA..

[22] Weiss AJ, Elixhauser A (2006). Overview of hospital stays in the United States, 2012: statistical brief #180.

[23] Farmer JE, Clark MJ, Sherman AK (2003). Rural versus urban social support seeking as a moderating variable in traumatic brain injury outcome. J Head Trauma Rehabil..

[24] Rudberg MA, Sager MA, Zhang J (1996). Risk factors for nursing home use after hospitalization for medical illness. J Gerontol A Biol Sci Med Sci..

[25] Ahmed A, Allman RM, DeLong JF (2003). Predictors of nursing home admission for older adults hospitalized with heart failure. Arch Gerontol Geriatr..

[26] Abate M, Schiavone C, Pelotti P, Salini V (2011). Limited joint mobility (LJM) in elderly subjects with type II diabetes mellitus. Arch Gerontol Geriatr..

[27] Feenstra J, Grobbee DE, Remme WJ, Stricker BH (1999). Drug-induced heart failure. J Am Coll Cardiol..

[28] Korkia P, Stimson GV (1997). Indications of prevalence, practice and effects of anabolic steroid use in Great Britain. Int J Sports Med..

[29] Schacke H, Docke WD, Asadullah K (2002). Mechanisms involved in the side effects of glucocorticoids. Pharmacol Ther..

[30] Wehling M (2014). Non-steroidal anti-inflammatory drug use in chronic pain conditions with special emphasis on the elderly and patients with relevant comorbidities: management and mitigation of risks and adverse effects. Eur J Clin Pharmacol..

[31] Whelton A (2000). Renal and related cardiovascular effects of conventional and COX-2-specific NSAIDs and non-NSAID analgesics. Am J Ther..

[32] Harirforoosh S, Jamali F (2009). Renal adverse effects of nonsteroidal anti-inflammatory drugs. Expert Opin Drug Saf..

[33] Khanna D, Fitzgerald JD, Khanna PP, Bae S, Singh MK, Neogi T (2012). 2012 American College of Rheumatology guidelines for management of gout. Part 1: systematic nonpharmacologic and pharmacologic therapeutic approaches to hyperuricemia. Arthritis Care Res (Hoboken).

[34] Primatesta P, Plana E, Rothenbacher D (2011). Gout treatment and comorbidities: a retrospective cohort study in a large US managed care population. BMC Musculoskelet Disord..

